# A dedicated vascular access clinic for children on haemodialysis: Two years’ experience

**DOI:** 10.1007/s00467-016-3428-z

**Published:** 2016-08-07

**Authors:** Rukshana Shroff, Rosalie B Sterenborg, Adam Kuchta, Andrew Arnold, Nicholas Thomas, Lynsey Stronach, Soundrie Padayachee, Francis Calder

**Affiliations:** 1 Great Ormond Street Hospital for Children NHS Foundation Trust, London, UK; 2 Guy’s and St Thomas’s Hospital NHS Foundation Trust, London, SE1 9RT UK

**Keywords:** Arteriovenous fistulae, Children, Vascular ultrasound, Vascular access clinic, Haemodialysis

## Abstract

**Background:**

Arteriovenous fistula (AVF) formation for long-term haemodialysis in children is a niche discipline with little data for guidance. We developed a dedicated Vascular Access Clinic that is run jointly by a transplant surgeon, paediatric nephrologist, dialysis nurse and a clinical vascular scientist specialised in vascular sonography for the assessment and surveillance of AVFs. We report the experience and 2-year outcomes of this clinic.

**Methods:**

Twelve new AVFs were formed and 11 existing AVFs were followed up for 2 years. All children were assessed by clinical and ultrasound examination.

**Results:**

During the study period 12 brachiocephalic, nine basilic vein transpositions and two radiocephalic AVFs were followed up. The median age (interquartile range) and weight of those children undergoing new AVF creation were 9.4 (interquartile 3–17) years and 26.9 (14–67) kg, respectively. Pre-operative ultrasound vascular mapping showed maximum median vein and artery diameters of 3.0 (2–5) and 2.7 (2.0–5.3) mm, respectively. Maturation scans 6 weeks after AVF formation showed a median flow of 1277 (432–2880) ml/min. Primary maturation rate was 83 % (10/12). Assisted maturation was 100 %, with two patients requiring a single angioplasty. For the 11 children with an existing AVF the maximum median vein diameter was 14.0 (8.0–26.0) mm, and the median flow rate was 1781 (800–2971) ml/min at a median of 153 weeks after AVF formation. Twenty-two AVFs were used successfully for dialysis, a median kt/V of 1.97 (1.8–2.9), and urea reduction ratio of 80.7 % (79.3–86 %) was observed. One child was transplanted before the AVF was used.

**Conclusions:**

A multidisciplinary vascular clinic incorporating ultrasound assessment is key to maintaining young children on chronic haemodialysis via an AVF.

## Introduction

Adequate vascular access is a key factor for successful haemodialysis (HD). The ideal vascular access, according to the National Kidney Foundation–Dialysis Outcomes Quality Initiative (NKF-DOQI) guidelines, should deliver an adequate flow rate in combination with durability and a low rate of complications [1]. An arteriovenous fistula (AVF) is generally considered to be the optimum access for HD in adults and, as suggested by an emerging body of evidence, also in children [2–4]. However, most children continue to have a cuffed central venous catheter (CVC) as the first choice for vascular access [5, 6].

CVCs allow ease of insertion, immediate use and needle-free dialysis, but they have several significant disadvantages, namely, they are frequently associated with infection, thrombosis and stenosis that may cause obstruction of the central vessels [2, 3, 6]. Although AVFs have a significant primary failure rate (up to 40 % failure rate reported in the adult literature [7] and 25 % reported in a paediatric study [2]) and long maturation time and require expert surgical skills, they are associated with lower infection rates, fewer hospitalisations and improved access longevity [2, 6, 8, 9]. In our centre, a retrospective review of AVF outcomes in the 2-year period before the opening of a dedicated Vascular Access Clinic showed that five of 25 (25 %) children had primary AVF failure (successful revision possible in 2 of 3), and three had secondary AVF failure (13.6 %) [2]. Learning from this experience and outcomes reported elsewhere [10–12], we set up a multidisciplinary Access Clinic; the the outcomes are reported here.

Published experience in the use of an AVF for long-term HD in children is sparse, and direct comparisons should not be drawn between the paediatric and adult populations [6, 7]. The vascular biology of children is different to that of adults; hence, the ideal vein and artery size, maturation times and expected volume flow rates to achieve a functioning fistula in children on HD are largely unknown [6]. In addition, fistula function is not the only factor in successfully dialysing a child on HD: a multidisciplinary approach involving the child, parents, physicians, dialysis nurses, play specialists and psychologists is essential to achieving high quality long-term dialysis [11].

In order to increase the use of AVFs in children on long-term HD, we developed a dedicated Vascular Access Clinic, run jointly by a transplant surgeon, paediatric nephrologist, dialysis nurse and clinical vascular scientist specialised in vascular sonography, to provide specialised care for the initiation and monitoring of AVFs in children. Here, we report our 2-year experience and outcomes.

## Methods

We established a specialist Vascular Access Clinic at Great Ormond Street Hospital, London in June 2013. Over the following 2 years, new AVFs were created in 12 children with end-stage renal disease, and another 11 children with pre-existing AVFs were followed up. This is a review of the prospectively collected data. Details of the study population are shown in Table 1.

**Table 1:** Demographics of the patient cohort

Patient characteristics	New AVF patients (*n* = 12)	Pre-existing AVF patients (*n* = 11)
Age AVF formed (years)	9.2 (6.7–14.5)	9.6 (3.0–17.0)
Weight AVF formed (kg)	27.9 (20.6–45.8)	26.9 (14.0– 62.9)
Gender (males)	8 (66.7)	5 (45.5)
Underlying renal disease		
Congenital anomalies of the kidneys and urinary tract (CAKUT)	9 (75)	9 (82)
Cystic kidney disease	2 (16.6)	–
Glomerulopathies	1 (8.3)	2 (18)
Access site		
Brachiocephalic	5 (41.7)	7 (63.6)
Radiocephalic	2 (16.6)	–
Basilic vein transposition	5 (41.7)	4 (36.7)
Stages operation		
One stage	7 (60)	7 (63.6)
Two stage	5 (40)	4 (36.7)
Angioplasty	2 (16.6)	0 (-)

Data are presented as the median with the interquartile range (IQR) in parenthesis, or as a number with the percentage in parenthesis, as appropriate

AVF, Arteriovenous fistulae

All 21 children treated in the Vascular Access Clinic during the 2-year study period were assessed clinically and by ultrasound examination by a clinical vascular scientist specilised in vascular sonography. Both examinations are considered to be essential and are complimentary but give different levels of detail regarding the vascular biology for AVF planning.

Children are seen before a HD session in a warm, child-friendly environment. With the child sitting upright, a clinical examination of both arms is performed. The quality of the pulse at the elbow and wrist is assessed by palpation—if there is a noticeable difference, then bilateral blood pressures are measured followed by an arterial duplex assessment. A simple tourniquet is applied just below the ante-cubital fossa when assessing the forearm veins and around the proximal humerus for assessment of the upper arm veins. In some patients, venodilation is augmented by eliciting a Lewis Response [13]—by firmly rubbing the skin overlying the vein of interest venodilatation is achieved. The relationship between the vein and artery is crucial in terms of the incision and amount of mobilization necessary to form the AVF. Drawing the positions of the vessels on the skin is a useful aid when the surgical technique is discussed with the parent and child. The chest is examined for evidence of distended neck veins and superficial subcutaneous venous dilatation indicative of central venous obstruction. If such an obstruction is present, a bilateral central venogram with intravenous contrast or magnetic resonance angiogram (MRA) may be performed (regardless of the presence of a CVC). However, we use a technique of non-contrast MRA, also called time-of-flight or inflow angiography, that utilizes phase differences to distinguish blood from static tissue, thus avoiding contrast agents and gadolinium.

**Fig. 1:**
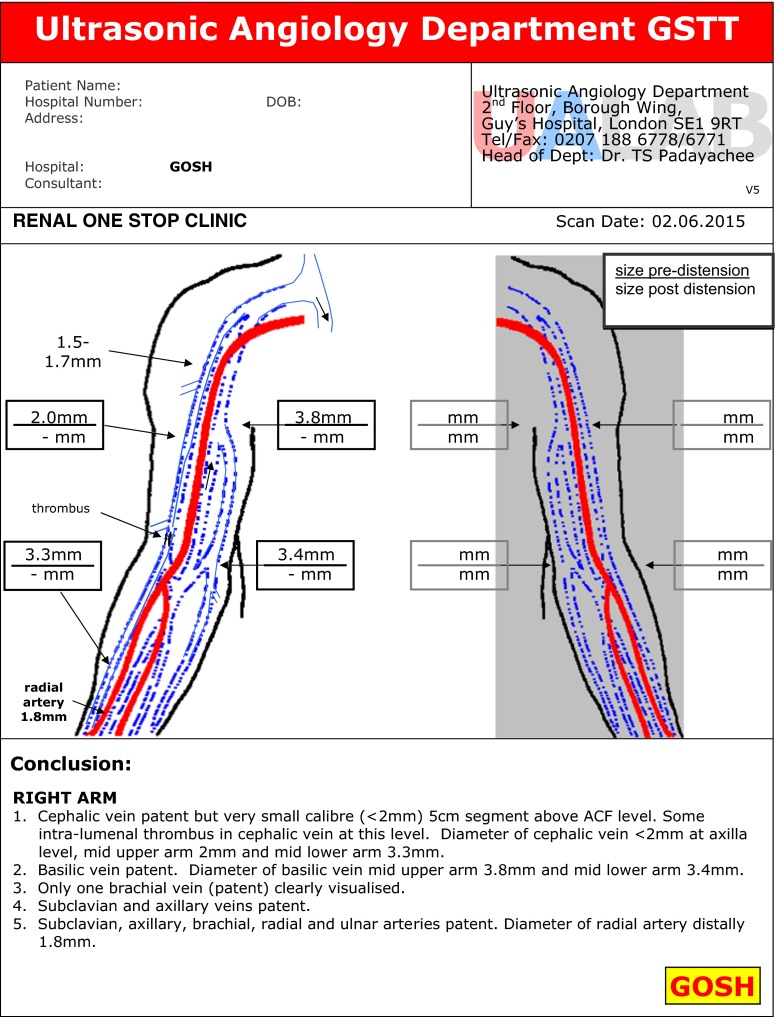
A typical mapping diagram showing ultrasound measurements of the arm vessels. *ACF* Antecubital fossa

The ultrasound assessment is performed in an adjacent room. Children are scanned in the supine position in a warm, child-friendly environment. The veins in both arms are mapped first without and the application of a simple tourniquet and then with a tourniquet. The arteries from the proximal brachial to the wrist are also mapped. Maximum vein and arterial diameters are measured. A typical mapping diagram is shown in Fig. 1. Since vein size can fluctuate depending on the room temperature, tourniquet application and the Lewis response, we always describe the maximum vein diameter and select the largest vein for AVF formation in each child, with a preference for distal (i.e. radial) over proximal vessels when possible.

Children are evaluated approximately 6 weeks after AVF formation to assess the maturation of the fistula by ultrasound examination [maximum vessel diameter (without tourniquet application) and volume flow are recorded] and clinical examination. All children enter a surveillance program consisting of volume flow assessment (by ultrasound examination off dialysis), clinical examination (including haemodynamic characteristics, overlying skin condition, dilatation of central veins on the chest wall, strength of distal pulses, symptoms of steal) and a review of dialysis adequacy, at regular intervals of 3–6 months.

The choice of AVF formed is dependent on a number of factors, including vessel anatomy, previous access history, hand dominance, intention for self cannulation and child/parental choice (particularly relevant for girls concerned with cosmesis). A single-stage cephalic-based AVF at the wrist or elbow is the first choice fistula; when this is not possible a brachiobasilic AVF is made in two stages.

Fistulas are needled by the rope ladder or button-hole technique. New AVFs are first needled by the rope ladder technique, progressing to button-hole needling in patients who self-needle or in whom a single carer consistently needles the fistula.

### Statistical analysis

Since most data were non-parametric, results are presented as the median and interquartile range (IQR). Due to the small number of patients only descriptive statistics were possible.

## Results

Over a 2-year period we managed 23 patients in our clinic, among whom 12 were new patients (pre-AVF creation) and 11 had pre-existing AVFs. During these 2 years of practice the percentage of children dialysing via a CVC decreased from 68 to 22 %.

### Types of AV fistulas

All fistulas were formed in the upper limb: brachiocephalic (*n* = 12), basilic vein transposition (*n* = 9) and radiocephalic (*n* = 2) fistulas. The median age and weight at AVF creation were 9.4 (IQR 3–17) years and 26.9 (IQR 14–67) kg, respectively. All children had started HD via a CVC; of these two children (16 %) were late presenters and had required immediate dialysis.

### New AVF patients

#### Vessel selection and AVF flow rate

Details of the AVF site and number of stages in AVF formation are shown in Table 1. Ultrasound mapping of the vein (with tourniquet) and artery used to create the AVFs showed a maximum median diameter of 3.0 (IQR 2–5) and 2.7 (IQR 2–5.3) mm, respectively.

At a median of 6 weeks after AVF formation, all children were assessed for AVF maturation. The maximum median vein (without tourniquet) and artery diameter was 8.6 (IQR 5.5–11) and 5.5 (IQR 4–8) mm, respectively. The median flow through the AVF was 1277 (IQR 432–2880) ml/min.

#### AVF functional outcomes

Fistulas were needled at a median of 8 (IQR 5.1–62) weeks after the final surgical procedure. Maturation is defined by clinical examination in combination with ultrasound assessment applying the KDOQI ‘rule of 6’s’. AVFs are first needled with size 17G needles, and depending on the vessel size and as tolerated by the child, the needles are increased to 14G or 15G. One child with cardiomyopathy and a very low blood pressure had delayed fistula maturation, but was successfully needled at 62 weeks without further intervention. In two children, extensive psychosocial preparation was required, thereby delaying the time to first needling and highlighting the importance of a multidisciplinary team with nursing and psychosocial support as part of the Vascular Access Clinic.

The primary maturation rate among our study population was 81 % (9/11 AVFs; the patient with cardiomyopathy and delayed maturation was excluded). The assisted maturation rate was 100 %. Two children required angioplasty to achieve maturation: both had brachiocephalic AVFs with post-anastomotic stenosis and poor volume flow. A single angioplasty was required in each case. One child received a cadaveric renal transplant and never used the AVF created although it was functionally mature based on the ultrasound and clinical examinations.

Dialysis through a new AVF was started with a single needle (17G), using one lumen of the CVC simultaneously to return the blood. Within 2 weeks dialysis with two needles was achieved in ten of the 12 with new AVFs, and the CVC was removed. The rope ladder technique was used initially for needling the new AVR, with progression to the button-hole technique when possible. Haematoma formation (‘blowing’) using the AVF was seen in 30 % of children upon HD initiation via the AVF but managed conservatively by elevation, non-occlusive compression and re-assurance. There were no cases where HD had to be terminated due to this problem.

At the 6-month follow-up (*n* = 11), maximum median vein diameter (without tourniquet) and artery diameter were 8.6 (IQR 5.8–27) and 6.0 (4.8–9.5) mm, respectively, with a median flow rate of 1441 (IQR 600–2880) ml/min At last review after a median of 8.6 months post AVF formation (*n* = 11), all children had achieved excellent dialysis adequacy with a median urea reduction ratio (URR) of 80.7 % (IQR 79–86 %) and Kt/V of 1.97 (IQR 1.77–2.95).

### Pre-existing AVF patients

Details of the AVF site and number of stages in AVF formation are shown in Table 1.

#### AVF functional outcomes

Four children received a kidney transplant during the study period; all had functional AVFs with good flow rates and no evidence of clinically significant stenosis. The 1-year primary patency was 100 %. At a median of 179 weeks after fistula formation, the median flow rate was 1556 (IQR 700–2600) ml/min. The maximum median fistula vein diameter was 13.5 (IQR 8–27) mm and median artery diameter was 6.0 (IQR 4.8–11) mm. The median URR was 82 % (76–87 %) and Kt/V was 2.2 (1.6–2.98).

### Access complications

Four significant complications were seen in those using AVFs: (1) an episode of thrombophlebitis in the collateral veins in a basilic vein AVF with proximal subclavian stenosis, managed with elevation and analgesia; (2) minor limb hypertrophy in a child with a left brachiocephalic AVF; (3) surgical aneurysmectomy and inflow reduction by anastamotic revision and distilization in a high flow brachiocephalic AVF with cephalic arch syndrome; (4) excision of a bleeding side branch in a brachiocephalic AVF with venous hypertension. HD was continued in all cases without the need for a CVC.

## Discussion

Renal transplantation is the first choice for all children with end stage renal disease (ESRD) requiring renal replacement therapy. Where HD is chosen for chronic dialysis, the literature shows that for most children this is performed via a CVC. Although more complicated to initiate, an AVF should be considered as the optimal method for vascular access in the absence of imminent transplantation. Previous reports have suggested that vein diameters of 1.5–2.5 mm are used to fashion AVFs in children [3, 14], there are no data on optimal vessel diameter and outcomes of AVF formation in children. It would not be appropriate to extrapolate from adult studies since the vessels of children, although smaller, are far less likely to be affected by diabetes and long-standing renal disease that cause medial calcification and vessel stiffness [15]. To the best of our knowledge, this paper is the first to describe vessel diameter and outcomes of AVF formation in children.

### Culture change regarding AVF usage

Achieving a high rate of AVF usage in a paediatric HD unit may require a ‘culture change’ amongst medical/nursing staff and children/parents away from the perceived convenience of a CVC [3, 6]. The establishment of dedicated Vascular Access Clinics has been vital in providing a focal point for education, assessment and on-going management of children on chronic HD. To assess this centralized service, children and parents often travel long distances, and it is therefore essential that a ‘one stop’ approach is honoured for convenience and to minimize disruption, especially when the patients are attending school. Part of the ‘popularity’ of CVC access is the unfamiliarity with the assessment, formation, needling and on-going management of AVFs in children. We have found that our Vascular Access Clinic serves as a hub for parents to see and discuss the benefits of an AVF with the access team and other children, while medical students, nurses and medical staff are able to observe children thriving from the good quality access and dialysis they provide. In the small, centralized world of paediatric HD, ideas spread and the culture is gradually changing. After 2 years of practice the percentage of children dialysing at our Vascular Access Clinic via a CVC has decreased from 68 to 22 %.

### Suitability for AVF formation

The creation of an AVF should be considered for any child who (1) is likely to need HD and (2) has no prospect of an early renal transplant. This approach encourages early referrals to the Vascular Access Clinic where the education of the child and parent can commence. Initial discussions centre around the nature of an AVF (how it works, the operation and advantages over a CVC), the importance of vein preservation for now and future AVFs, and a review of other renal replacement options, including peritoneal dialysis and transplantation. Vascular imaging and patient examination take place in adjacent rooms so that the results of the clinical examination and real-time ultrasound imaging can be correlated. The child then meets with a dialysis specialist nurse and receives a DVD about undergoing HD via an AVF with further information and a contact phone number. After the AVF is created, the child is brought back to the clinic in 4 weeks to assess AVF maturation, identify and manage any complications and plan for needling of the AVF. Psychosocial support is arranged where required.

In young girls the position of an AVF is discussed at the initial clinic visit. For cosmesis, we have found a brachiocephalic AVF is often requested as it can be hidden under a sleeve. Hence, a brachiocephalic AVF in the dominant limb is commonly preferred to a basilic vein transposition in the non-dominant limb, the latter involving a larger incision and two-stage approach. The button-hole technique is advocated for needling the mature fistula to avoid aneurysmal dilatation.

### AVF maturation

A review of the literature revealed that vessel selection varies between centres [2, 4, 8–10, 16, 17], but the authors of these studies scarcely commented on primary failure and assisted maturation rates. We found that selecting veins with a maximal median diameter of 3 mm (with tourniquet application) and arteries with a maximum median diameter of 2.7 mm resulted in a primary maturation rate of 82 %. We would emphasize that clinical assessment is equally important as ultrasound findings in terms of selecting the appropriate vein. The 19 % (2 cases) of AVFs that failed primary maturation were rescued with a single balloon angioplasty for single post anastomotic lesions.

Of the 12 children with new AVFs, 11 were successfully cannulated for dialysis at a median of 8 weeks. The timing of initial cannulation reflects a number of factors, including application of the KDOQI ‘rule of 6’s’ [1, 3], availability of experienced staff to initiate cannulation, patient choice and clinical urgency. We tend to delay initiating dialysis until we are confident that the child is psychologically ready and the AVF is highly likely to function without complication. In distinction to adults, ‘prolonged maturation’ is a feature of the AVF of pediatric patients and reflects the differing vascular biology of this latter patient group. The vein size of the fistual and AVF flow rates are reviewed at 6 weeks to assess maturity. If the AVF is immature we will continue the ultrasound and clinical assessments at 6-week intervals until the fistula size is ‘static’ (no change in 6 weeks) before considering whether further intervention is necessary to achieve maturity. This approach does mean that the CVC may be retained for a longer time, but with the anticipation of a compliant child and functional AVF. Little data are available on angioplasty-assisted maturation of paediatric AVF, and we are reluctant to intervene until it is essential. Hand-ball squeeze exercises are prescribed for the older child to aid maturation.

For a brachiobasilic AVF we favour a two-stage approach as this allows the fistula vein to increase in diameter and length while it remains in situ, before making the transposition and superficialization. A clinically significant post-anastamotic stenosis, if it develops, is easier to surgically revise at the second stage when mobilizing the entire vein for the first time. Evidence favouring this approach is not substantiated in the literature, but is based almost entirely on adult studies [18].

### Volume flow

Surprisingly, the volume flow rates seen after AVF formation in relatively small vessels is comparable to those in adults. Paediatric vascular biology differs from that in adults [19, 20]; consequently, key features such as smaller but compliant vessels, short duration of uraemia, prolonged maturation, absence of steal syndrome and intolerance of central catheters are important considerations when dealing with the paediatric population. Similar flows have been reported by other research groups without complication [21].

We have observed that children’s vessels behave differently at surgery compared to those of adults. Besides the obviously smaller vessel size in children, paediatric vessels exhibit intense vasospasm when handled during surgery, which is not noted in adults. Paediatric vessels are also far less likely to be affected by diabetes and long-standing renal disease that cause medial calcification and vessel stiffness [15]. In addition, as stated in the section AVF maturation, distinct from the situation in adults, prolonged maturation is a feature of the paediatric AVF, possibly also due to the growth potential in children.

### Access complications

The four access complications which developed in our study population were all managed without the need to implant a CVC with its inherent problems. For those requiring surgical revision, a ‘single needle’ technique was used for HD while the post-operative swelling resolved.

### CVC usage

All patients commenced their HD programme with a CVC. This is far from the ideal situation—the literature on AVFs in adults shows a significantly increased mortality when HD is initiated with a CVC even if the patient is converted to an AVF later [22]. The reasons for failure to commence HD with a functional AVF relate to a late referral pattern, the need for visits to the Vascular Access Clinic at 6-week intervals, limited availability of operating time and a need for immediate dialysis in the ‘crash landers’. In the future, children who are likely to need chronic haemodialysis (i.e. no living donor identified for early transplantation and those for whom peritoneal dialysis is not an option) will be seen at an earlier stage in the course of their renal decline for education, vein preservation and access planning. The benefits of a multidisciplinary vascular clinic in access planning has been shown by another group [23]. Other paediatric groups who extoll AVF usage have also shown a reduction in CVC usage [24].

### Transplantation and AVF

Transplantation is unquestionably the best long-term renal replacement therapy for children in terms of survival, quality of life and cost. However, even following transplantation, we take the view that the AVF should not be ligated as it is a precious resource for the future if dialysis should become necessary once more. Earlier work by our group showed that 39 % of transplanted children (particularly teenagers) will return to dialysis during their childhood [25]. In addition, suitably trained staff can use the AVF for venesection, thereby maintaining the venous reserve for future AVF formation.

### Surveillance and monitoring

All AVFs are monitored by the patient and nursing staff by clinical assessment before the start of a dialysis session, and all patients are encouraged to seek early assistance in the case of concerns. A programmed assessment of volume flow occurs every 3–6 months in the access clinic by off-dialysis ultrasound (children travelling from further afield are seen every 6 months). A 25 % reduction from baseline flow is considered to be significant and warrants further investigation. Other units employ an ultrasound dilution technique, and this technique has been shown to be more sensitive than fistulograms for detecting haemodynamically significant stenosis [21].

### Long-term patency

We have seen 100 % primary patency over 3 years in the prevalent AVFs which is a testimony to the quality of care of the dialysis staff. These children are often the most difficult to transplant and hence face a prolonged and uncertain period on HD. The waiting time for first cadaveric transplant in the UK is often more than 1 year for children and increasing. Consideration for fistula formation should occur early in the assessment of the child with ESRD.

In conclusion, we have shown that a multidisciplinary vascular clinic incorporating ultrasound assessment is key to successful AVF formation and outcomes in young children.
